# New onset diabetes mellitus and cardiovascular events in Korean patients with acute myocardial infarction receiving high-intensity statins

**DOI:** 10.1186/s40360-021-00476-z

**Published:** 2021-02-04

**Authors:** Jah Yeon Choi, Cheol Ung Choi, Byoung Geol Choi, Yoonjee Park, Dong Oh Kang, Won Young Jang, Woohyeun Kim, Jin Oh Na, Jin Won Kim, Eung Ju Kim, Seung-Woon Rha, Chang Gyu Park, Hong Seog Seo, Myung Ho Jeong, Sung-Chull Chae, In-Whan Seong, Chang-Hwan Yoon, Kwang Soo Cha, Seok Kyu Oh, KAMIR-NIH Investigators, Woong Chol Kang, Seung Woon Rha, Kwang-Soo Cha, Hyeon Cheol Gwon, Seok Kyu Oh, Jei Keon Chae, Kyung-Kook Hwang, Chong Jin Kim, Jung Han Yoon, Jin Yong Hwang, Doo Il Kim, Seung Jae Joo

**Affiliations:** 1 Cardiovascular Center, Korea University Guro Hospital, 80, Guro-dong, Guro-gu, Seoul, 152-703 Republic of Korea; 2 Cardiovascular Center, Catholic University of Korea St. Vincent Hospital, Suwon, Republic of Korea; 3 Chonnam National University Hospital, Gwangju, Republic of Korea; 4 Kyungbook National University Hospital, Kyungbook National University School of Medicine, Daegu, Republic of Korea; 5 Chungnam National University Hospital, Daejeon, Republic of Korea; 6 Seoul National University Bundang Hospital, Seongnam, South Korea; 7 Pusan National University Hospital, Busan, South Korea; 8 Wonkwang University School of Medicine, Iksan, Korea

**Keywords:** Acute myocardial infarction, New-onset diabetes mellitus, Cardiovascular outcome, Atorvastatin, Rosuvastatin

## Abstract

**Background:**

High-intensity statin therapy is typically used in patients with acute myocardial infarction (AMI) for secondary prevention. However, there have been consistent concerns regarding its association with diabetes mellitus. We investigated the effect of high-intensity atorvastatin and rosuvastatin on new-onset diabetes mellitus (NODM) and cardiovascular outcomes over a 3-year follow-up period.

**Methods:**

Data from the Korea Acute Myocardial Infarction Registry were collected from November 2011 to October 2015, and 13,104 patients with AMI were enrolled from major cardiovascular centers. Among them, 2221 patients without diabetes who had been administered with high-intensity atorvastatin (40–80 mg) and rosuvastatin (20 mg) were investigated. The atorvastatin and rosuvastatin groups were evaluated for the incidence of NODM and major adverse cardiac events (MACE) including death, myocardial infarction, and revascularization cases in the following 3 years.

**Results:**

Baseline characteristics were comparable between the two groups. Event-free survival rate of NODM was not significantly different between the atorvastatin and rosuvastatin groups (92.5% vs. 90.8%, respectively; Log-rank *P*-value = 0.550). The event-free survival rate of MACE was also not significantly different between atorvastatin and rosuvastatin groups (89.0% vs. 89.6%, respectively; Log rank P-value = 0.662). Multivariate Cox analysis revealed that statin type was not a prognostic factor in the development of NODM and MACE.

**Conclusions:**

Administering high-intensity atorvastatin and rosuvastatin in patients with AMI produced comparable effects on NODM and clinical outcomes, suggesting their clinical equivalence in secondary prevention.

**Supplementary Information:**

The online version contains supplementary material available at 10.1186/s40360-021-00476-z.

## Background

Statins typically prevent cardiovascular events by lowering total and low-density lipoprotein (LDL) cholesterol levels in the serum. Considering their rapid and sustained clinical advantages, the current guideline recommends administration of high-intensity statins in patients with acute myocardial infarction (AMI) for secondary prevention. However, there has been consistent concern regarding its association with new-onset diabetes mellitus (NODM) [1]. Clinical trials, meta-analyses of randomized controlled trials (RCTs), and observational studies have demonstrated a 10–12% increase in NODM among patients receiving statins [2, 3]. Meta-analysis of five large-scale trials comparing intensive and moderate doses of statins have demonstrated that the risk of NODM further increases in intensive therapy groups [4]. However, it is unclear if the diabetogenic effect of statins is a class effect. Considering their crucial role of secondary prevention in patients with AMI, it would be important to identify the diabetogenic and cardioprotective effects of high-intensity statins. In Korea, atorvastatin 40–80 mg and rosuvastatin 20 mg are currently available as high-intensity statins for clinical use. Here, we investigated the effect of high-intensity atorvastatin and rosuvastatin on cardiovascular outcomes and NODM in patients with AMI over a 3-year follow-up period.

## Methods

### Data collection and study population

The Korea Acute Myocardial Infarction Registry (KAMIR), a Korean prospective, multicenter, nationwide database supported by the Korean Society of Cardiology, reflects real-world clinical practices of AMI patients in Asian. Twenty university or community hospitals have participated in the registry. Data collection at each institution level is performed by a study coordinator using a standardized case report form and the collected data are managed using web-based systems. The study was approved by the ethics committee at each institution. All the patients enrolled the study provided written informed consent.

**Fig. 1:**
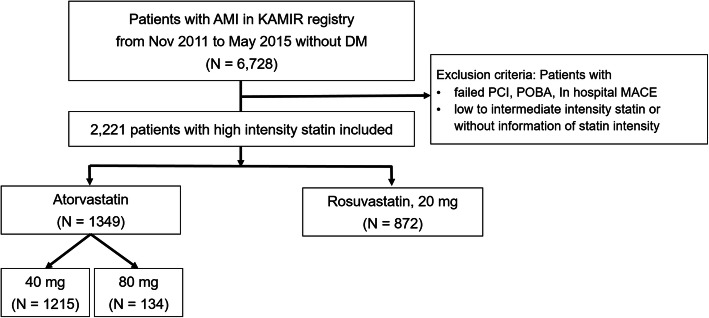
Patient flow chart. AMI: Acute myocardial infarction, DM: diabetes mellitus, KAMIR: Korea Acute Myocardial Infarction Registry, MACE: major adverse cardiac events, PCI: percutaneous coronary intervention, POBA: plain old balloon angioplasty

A total of 13,104 patients with AMI were enrolled in the KAMIR registry from November 2011 to October 2015. Among them, 6728 patients without a diagnosis of diabetes mellitus (DM) at enrollment, with a successful percutaneous coronary intervention (PCI) with drug-eluting stent implantation and high-intensity statin treatment were eligible for our study. Patients were selected considering the following exclusion criteria: history of DM or initial HbA1c level ≥ 6.5%, PCI with bare metal stent implantation or plain old balloon angioplasty. Additionally, we did not include patients with failed PCI, or in-hospital major adverse cardiac events (MACE). Finally, 2221 patients with AMI treated with high-intensity atorvastatin or rosuvastatin, according to 2014 ACC/AHA Release Updated Guideline, were analyzed in the study. Of them, 60.7% (1349/2221) of patients had received 40–80 mg atorvastatin and 39.3% (872/2221) had received 20 mg rosuvastatin (Fig. 1).

### Clinical outcome and definition

The primary endpoint was the incidence of NODM and the incidence of MACE during the 3 years of clinical follow-up. Secondary endpoints were each component of MACE, reasons for mortality, MI, and revascularization. NODM was defined as an HbA1c level ≥ 6.5% or new administration of oral hypoglycemic agents. The patients’ clinical data were obtained by face-to-face interviews during regular outpatient visits, medical chart reviews, and telephonic interviews.

### Statistical analysis

For continuous variables, differences between the two groups were analyzed by Student’s t-test. Data were presented as mean ± standard deviation (SD). For discrete variables, differences were analyzed using the Chi-square test or Fisher’s exact test and presented as counts and percentages. The cumulative incidence of NODM and MACE was calculated using the Kaplan–Meier method, and the intergroup differences were analyzed using the log-rank test. Cox-proportional hazard models reporting hazard ratio (HR) and 95% confidence interval (CI) was performed to identify potential prognostic factors for NODM and MACE. For multivariate analysis, variables with significant *P*-values (< 0.05) in the univariate analysis were included. For all analyses, a P-value < 0.05 was considered statistically significant. The data were analyzed using SPSS (version 22.0, Inc. Chicago, IL).

## Results

### Baseline characteristics

Baseline clinical, laboratory, and angiographic characteristics are demonstrated in Table 1. We did not observe any significant intergroup differences with regard to age, gender, LV systolic function, incidence of STEMI, and underlying diseases such as hypertension and cerebrovascular accidents. Patients in the rosuvastatin group had higher levels of LDL cholesterol and peak CK-MB and a longer total stent length than those in the atorvastatin group. Dual antiplatelet therapy (DAPT) rate was above 99% in both groups; however, the composition of DAPT was different in that the prescription rate of prasugrel was higher in the atorvastatin group and that of ticagrelor was higher in the rosuvastatin group. Numbers of patients taking ACEi or ARB and β blockers were higher in the atorvastatin group than in the rosuvastatin group.

**Table 1:** Baseline clinical characteristics and angiographic and procedural characteristics

Variables	Atorvastatin (*n* = 1349)	Rosuvastatin (*n* = 872)	*P*-value
Men	1105 (81.9%)	720 (82.6%)	0.693
Age (years)	61.0 ± 12.5	61.0 ± 12.6	0.918
LV ejection fraction (%)	54.1 ± 9.9	54.0 ± 9.6	0.906
Body mass index (kg/m^2^)	24.5 ± 3.2	24.4 ± 3.4	0.442
Myocardial infarction			
ST-segment elevation	719 (53.3%)	478 (54.8%)	0.483
Non-ST-segment elevation	630 (46.7%)	394 (45.2%)	
Hypertension	549 (40.7%)	329 (37.7%)	0.162
Cerebrovascular accidents	42 (3.1%)	29 (3.3%)	0.781
Total cholesterol (mg/dl)	195.5 ± 42.0	196.6 ± 45.6	0.563
Triglyceride (mg/dl)	146.5 ± 120.3	145.4 ± 139.1	0.852
HDL-cholesterol (mg/dl)	44.1 ± 11.1	44.0 ± 11.1	0.790
LDL-cholesterol (mg/dl)	126.5 ± 36.3	130.6 ± 40.3	0.020
CK-MB (mg/dl)	125.2 ± 159.2	139.9 ± 145.1	0.029
Glucose (mg/dl)	136.7 ± 37.4	138.8 ± 37.9	0.216
Creatinine (mg/dl)	0.97 ± 0.94	0.94 ± 0.42	0.340
**Discharge medications**			
Aspirin	1343 (99.6%)	865 (99.2%)	0.280
Clopidogrel	855 (63.4%)	473 (54.2%)	< 0.001
Prasugrel	188 (13.9%)	68 (7.8%)	< 0.001
Ticagrelor	302 (22.4%)	325 (37.3%)	< 0.001
Cilostazol	57 (4.2%)	29 (3.3%)	0.283
Calcium channel blockers	63 (4.7%)	45 (5.2%)	0.600
β blockers	1181 (87.5%)	714 (81.9%)	< 0.001
ACEi	687 (50.9%)	321 (36.8%)	< 0.001
ARB	426 (31.6%)	336 (38.5%)	0.001
**Procedural characteristics**			
Total stent length (mm)	27.6 ± 12.2	39.8 ± 13.4	< 0.001
Total stent number	1.17 ± 0.41	1.20 ± 0.45	0.077
Stent diameter (mm)	3.20 ± 0.44	3.14 ± 0.44	0.062

*ACEi* angiotensin-converting enzyme inhibitor, *ARB* angiotensin receptor blocker, *CK-MB* Creatine Kinase-MB, *HDL* high-density lipoprotein, *LDL* low-density lipoprotein, *LV* left ventricle

### Clinical outcomes

**Fig. 2:**
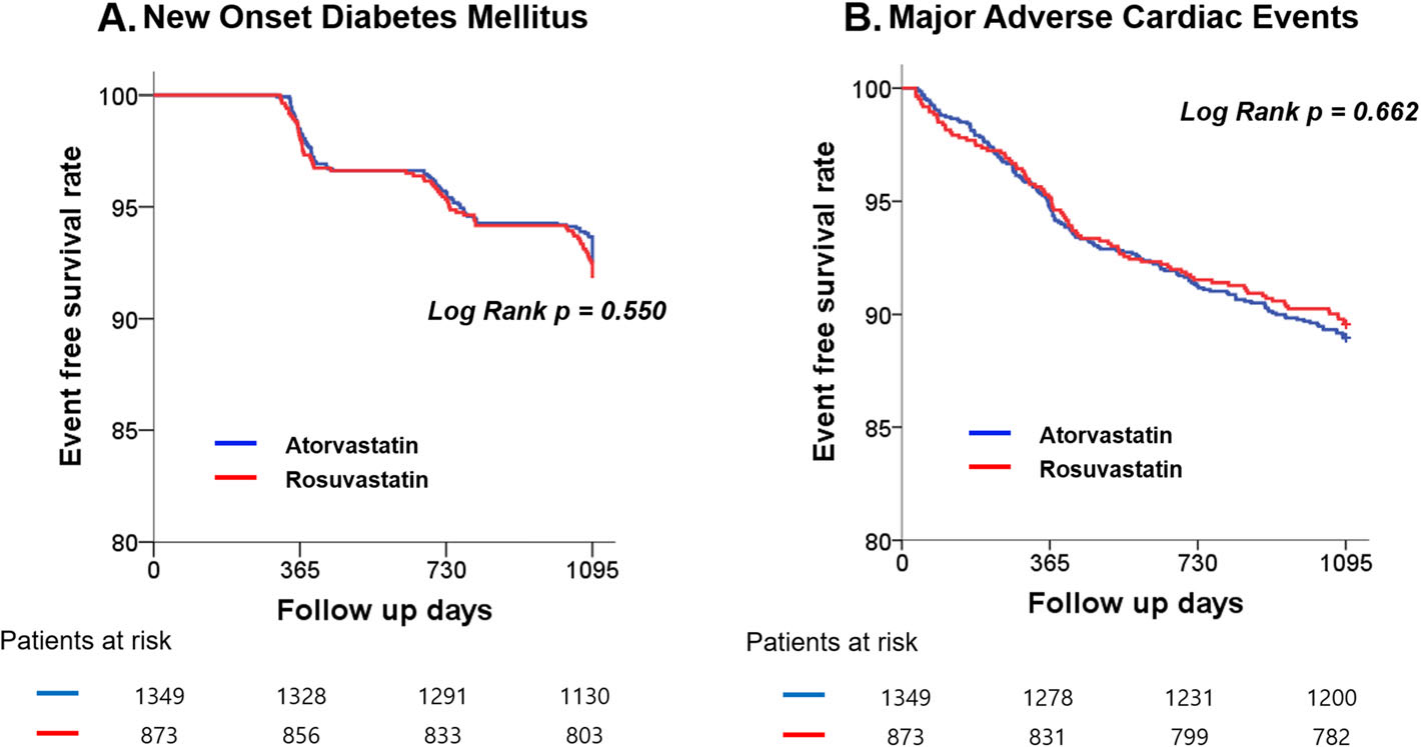
Kaplan–Meier curves for event-free survival rate of new-onset diabetes mellitus (**a**) and major adverse cardiac events (**b**) according to statin type

The cumulative incidence of NODM up to 3 years using Kaplan-Meier method is presented in Fig. 2a and Table 2. There was no significant difference in the event-free survival rate of NODM between the atorvastatin and rosuvastatin groups (92.5% vs. 90.8%, respectively; Log-rank *P*-value = 0.550). Kaplan–Meier curves for the cumulative incidence of MACE up to a period of 3 years are presented in Fig. 2b and Table 2. There was no significant difference between the atorvastatin and rosuvastatin groups regarding the event free survival rate of MACE (89.0% vs. 89.6%, respectively; Log rank P-value = 0.662), reasons for mortality, myocardial infarction, and revascularizations. Comparing 40 mg and 80 mg of atorvastatin groups with 20 mg of rosuvastatin group revealed no significant differences in the event-free survival rate of NODM and MACE (see Additional file 1: Fig. S1A and B).

**Table 2:** Cardiovascular Outcomes at 3 Years

Clinical outcome	Atorvastatin (*n* = 1349)	Rosuvastatin (*n* = 872)	*P*-value
New onset diabetes mellitus	99 (7.5)	70 (9.2)	0.550
MACE	149 (11.0)	91 (10.4)	0.662
All cause of mortality	49 (3.6)	25 (2.9)	0.335
Cardiac death	19 (1.4)	14 (1.6)	0.710
Non-cardiac death	30 (2.2)	11 (1.3)	0.103
Any myocardial infarction	30 (2.3)	15 (1.8)	0.409
STEMI	8 (0.6)	1 (0.1)	0.083
NSTEMI	22 (1.7)	14 (1.6)	0.956
Any revascularization	98 (7.4)	62 (7.2)	0.892
TLR	24 (1.9)	21 (2.5)	0.312
TVR	48 (3.7)	34 (4.0)	0.692
NTVR	51 (3.9)	31 (3.7)	0.788
Stroke	27 (2.0)	10 (1.2)	0.127

*MACE* major adverse cardiovascular event, *STEMI* ST-segment elevation myocardial infarction, *NSTEMI* Non-ST-segment elevation myocardial infarction, *NTVR* non-target vessel revascularization, *TLR* target lesion revascularization, *TVR* target vessel revascularization

Potential prognostic factors for NODM were identified via univariate Cox regression analysis. Higher random glucose and triglyceride levels were both significant prognostic factors for NODM in univariate and multivariate analysis. However, the type of statin used was not (HR = 1.098, 95% confidence interval [CI]: 0.808–1.491, *P* = 0.551, Table 3). Conventional risk factors including older age, lower left ventricular ejection fraction (LVEF), and higher creatinine levels were associated with a higher incidence of MACE. Use of new antiplatelet agents such as ticagrelor or prasugrel was a significant prognostic factor in univariate analysis, not however, in the multivariate analysis. The type of high-intensity statin, atorvastatin or rosuvastatin, was not a potential prognostic factor for MACE (HR = 0.944% confidence interval [CI]: 0.727–1.225, *P* = 0.662, Table 3).

**Table 3:** Cox regression of clinical outcome

	Univariable		Multivariable	
	Hazard ratio (95% CI)	*p* value	Hazard ratio (95% CI)	*P*-value
**NODM**				
Age	0.988 (0.976–1.000)	0.052		
Male gender	1.307 (0.848–2.013)	0.225		
Glucose	1.009 (1.006–1.011)	< 0.001	1.009 (1.006–1.011)	< 0.001
Triglyceride	1.001 (1.000–1.002)	0.011	1.001 (1.000–1.002)	0.021
β blocker	0.768 (0.517–1.139)	0.189		
Statin type	1.098 (0.808–1.491)	0.551		
**MACE**				
Age	1.026 (1.016–1.037)	< 0.001	1.020 (1.009–1.032)	0.001
Male gender	0.964 (0.695–1.336)	0.824		
LVEF	0.974 (0.961–0.986)	< 0.001	0.980 (0.966–0.993)	0.003
Glucose	1.003 (1.000–1.006)	0.089		
Creatinine	1.155 (1.080–1.235)	< 0.001	1.204 (1.062–1.364)	0.004
LDL cholesterol	0.995 (0.992–0.999)	0.011	0.998 (0.994–1.002)	0.258
Statin type	0.944 (0.727–1.225)	0.662		
Ticagrelor or prasugrel	0.685 (0.522–0.899)	0.006	0.784 (0.585–1.051)	0.104
ACEi or ARB	0.761 (0.569–1.018)	0.066		
β blocker	0.855 (0.609–1.201)	0.367		

*ACEi* angiotensin-converting enzyme inhibitor, *ARB* angiotensin receptor blocker, *LDL* low-density lipoprotein, *LVEF* left ventricle ejection fraction

## Discussion

To the best of our knowledge, this is the first study to investigate the effect of high-intensity statin treatment on the development of NODM and MACE in Korean patients with AMI. Our results indicated that high-intensity atorvastatin and rosuvastatin therapies showed no significant difference with regard to the incidence of NODM and cardiovascular events.

Statins reduce serum LDL cholesterol level and the risk of cardiovascular events. As numerous studies revealed that the degree of cardiovascular risk reduction is proportional to the statin intensity [5, 6], the current guidelines strongly recommend high-intensity or maximally tolerated intensity statin therapy in patients with AMI in the absence of contraindications [7, 8]. However, several studies have suggested that statins increase the incidence of NODM [3, 9]. The issue has started attracting attention since the Justification for the Use of Statin in Prevention: An Intervention Trial Evaluating Rosuvastatin (JUPITER) trial revealed a higher incidence of NODM in rosuvastatin treated patients for primary prevention than in patients with placebo [10]. Numerous observational studies [11, 12] and meta-analyses of major RCT [3, 13] have consistently reported an increased incidence of NODM in patients treated with statin. In Korea, a population-based cohort study using the Korean National Health Insurance claims database has shown an increased incidence of NODM in statin-treated groups [2].

Whether the diabetogenic effect of statin is a class effect has been a controversial subject. Typically, atorvastatin and rosuvastatin are thought to unfavorably influence glycemic parameters, while pitavastatin and pravastatin have relatively neutral effects on glycemic control regardless of the presence or absence of DM [1]. Our group recently published a report regarding the favorable glycemic effects of moderate-intensity pitavastatin in comparison to those of moderate-intensity atorvastatin and rosuvastatin in patients with AMI [14]. Despite the current guidelines recommending high-intensity or maximally tolerated statin for secondary prevention in patients with AMI [7, 8], there is no study, to the best of our knowledge, that has compared the diabetogenic effects of different high-intensity statins.

Several studies were conducted regarding the cardiovascular outcomes after high-intensity statin therapy. In patients with acute coronary syndrome, both the atorvastatin and rosuvastatin groups had comparable effects on lipid parameters [15, 16], although patients with familial hypercholesterolemia in the rosuvastatin group demonstrated a greater reduction in LDL cholesterol levels than those in the atorvastatin group [17]. Some studies have reported more favorable effects of rosuvastatin on reducing atherosclerotic plaque volume [15, 18] and plaque stabilization [15] than of atorvastatin; however, there has been no significant difference with regard to the cardiovascular outcome in both groups [17, 19]. In line with these previous studies, we could not identify the differences between the effects of high-intensity atorvastatin and rosuvastatin administration on major cardiovascular events.

Several studies have suggested the possible mechanisms underlying the effect of statin on glucose metabolism. Some studies have suggested the interconnection between glucose and lipid metabolisms by demonstrating gene variants affecting glucose metabolism [20–22], cholesterol-dependent conformational change in glucose transporter protein [23], or deleterious effect on islet β cells [24] by statins. There is a scarcity of data regarding the mechanisms underlying the different diabetogenic effects of statins; hence, further study would be needed.

This study has several limitations. First, our study is not an RCT, which inevitably leads to selection bias and an imbalance in baseline characteristics. However, as KAMIR is a prospective registry that enrolls Korean patients with AMI, it can represent real-word clinical data. Second, there is a lack of data on rosuvastatin 40 mg and a relatively small number of patients have been treated with atorvastatin 80 mg; hence, dose-dependent increases in NODM could not be demonstrated in our study. Third, this study lacks data regarding the compliance of statin during the follow-up period. Most conventional variables such as age, LVEF, renal function, and new antiplatelet agents were shown to be significant prognostic factors for cardiovascular outcomes, but RAS blockers and β blockers were reported as modest and insignificant prognostic factors, respectively. Despite its limitations, to the best of our knowledge, this is the first study from a multicenter registry that demonstrated detailed real-world data on the effect of high-intensity statin on incidence of NODM and MACE in patients with AMI.

## Conclusions

In conclusion, high-intensity atorvastatin therapy showed similar incidence of NODM and cardiovascular events when compared with high-intensity rosuvastatin therapy in patients with AMI. Although prospective, randomized trials with a larger study population are needed to clarify our results, the outcomes presented here provide supportive evidence for the diabetogenic and cardioprotective effects of high-intensity statins in patients with AMI.

## Supplementary Information

**Additional file 1.**

## Data Availability

The datasets generated and/or analysed during the current study are not publicly available because it is confidential, but are available from professor Myung Ho Jeong on reasonable request.

## Abbreviations

AMI: acute myocardial infarction
KAMIR: Korea Acute Myocardial Infarction Registry
LDL: low-density lipoprotein
MACE: major adverse cardiac events
NODM: new-onset diabetes mellitus
PCI: percutaneous coronary intervention
RCT: randomized controlled trial
