# Supplementary material for: New onset diabetes mellitus and cardiovascular events in Korean patients with acute myocardial infarction receiving high-intensity statins
Source: BMC Pharmacol Toxicol. 2021 Feb 4;22:11. doi: 10.1186/s40360-021-00476-z (PMC7863364; doi:10.1186/s40360-021-00476-z)

**Additional file 1**

Fig. S1. Kaplan–Meier curves for event-free survival rate of new-onset diabetes mellitus (A) and major adverse cardiac events (B) according to statin type and dose


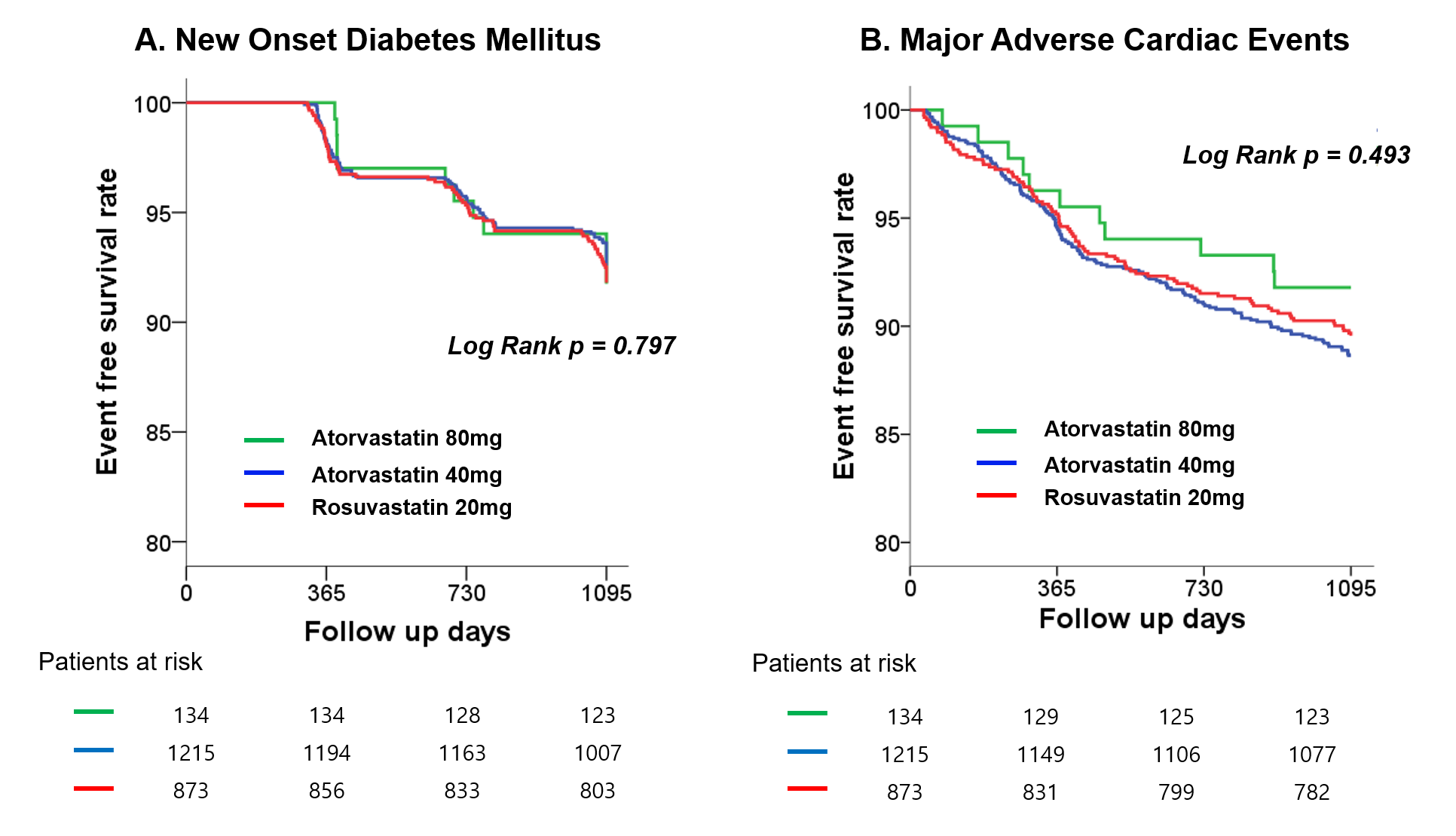

Supplement: Supplementary file 1 — Additional file 1. [file 40360_2021_476_MOESM1_ESM.docx]
